# The *WOX* Genes from the Intermediate Clade: Influence on the Somatic Embryogenesis in *Medicago truncatula*

**DOI:** 10.3390/plants13020223

**Published:** 2024-01-13

**Authors:** Daria V. Yakovleva, Elena P. Efremova, Kirill V. Smirnov, Veronika Y. Simonova, Zakhar S. Konstantinov, Varvara E. Tvorogova, Ludmila A. Lutova

**Affiliations:** 1 Department of Genetics and Biotechnology, Saint Petersburg State University, 7/9 Universitetskaya emb, Saint Petersburg 199034, Russia; enginequs@gmail.com (D.V.Y.); efremova.bio@gmail.com (E.P.E.); la.lutova@gmail.com (L.A.L.); 2 All-Russia Research Institute for Agricultural Microbiology, Podbelsky Chausse 3, Pushkin, Saint Petersburg 196608, Russia; kirill.vad.smirnov@gmail.com; 3 Plant Biology and Biotechnology Department, Sirius University of Science and Technology, 1 Olympic Avenue, Sochi 354340, Russia; nikasimonova14@gmail.com (V.Y.S.); zakhar.konstantinov25@gmail.com (Z.S.K.); 4 Center for Genetic Technologies, N. I. Vavilov All-Russian Institute of Plant Genetic Resources (VIR), 42 Bolshaya Morskaya Street, Saint Petersburg 190000, Russia

**Keywords:** somatic embryogenesis, *WOX* genes, legumes, *Medicago truncatula*, morphogenic regulators

## Abstract

Transcription factors from the WOX family are well-known regulators of cell proliferation and differentiation in plants. Herein, we focused on several *WOX* genes from the intermediate clade and checked their impact on somatic embryogenesis using the model legume object *Medicago truncatula*. As a result, we show that *MtWOX9-1* overexpression not only stimulates somatic embryogenesis in the embryogenic *M. truncatula* line, as it was shown previously, but can also induce somatic embryogenesis in the non-embryogenic line. Other intermediate clade WOX, including the close paralog of MtWOX9-1, as well as WOX11 homologs, did not have any significant impact on somatic embryogenesis in our in vitro cultivation system. Together, our results give new information about the diversity of the WOX family proteins and their specific functions. These data can be used for the search of new regeneration stimulators.

## 1. Introduction

The WOX (WUSCHEL-LIKE HOMEOBOX) transcription factors are homeodomain-containing proteins with a conserved DNA-binding domain. Members of this family have many different functions in the regulation of meristem activity, regeneration, embryogenesis, and other processes related to cell proliferation and differentiation. According to the phylogenetic analysis, the *WOX* gene family can be divided into three large clades, including modern (T3 type, or WUS), intermediate (T2 type), and ancient (T1 type) clades. Members of the modern clade (*WUS*, *WOX1*–*7* in *Arabidopsis thaliana* and their orthologs in other plant species) regulate apical, lateral, and leaf meristem functions and are also involved in embryogenesis. The ancient clade includes genes *WOX10*, *13*, *14*, and their orthologs, which have more divergent and less specific functions. Members of the intermediate clade, i.e., *WOX8*, *9*, *11*, and *12*, and their orthologs, are usually associated with the embryogenesis process, formation of adventitious roots, and regeneration as a whole [1].

The intermediate clade can also be divided into two subclades: WOX8/9 and WOX11/12 [2]. Members of the WOX11/12 subclade are primarily involved in adventitious roots and/or callus formation. In *A. thaliana*, after plant organ wounding, the auxin signaling pathway activates the expression of *WOX11* in the future root founder cell [3]. In its turn, WOX11 triggers the expression of other genes (*LBD16*, *WOX5*, and *WOX7*), which initiate the generation of adventitious roots and/or calli [4,5]. Inhibition of the WOX11 pathway results in decreased rooting ability, whereas overexpression of *WOX11* increases the ability of detached leaves to form roots [3]. The regulation of adventitious root initiation in leaves or in damaged roots by the WOX11 transcription factor or its orthologs has been studied in many species, including *Panax* [6], *Malus* [7], *Populus* [8], and *Oryza* [9] genera. In *Panax ginseng* adventitious roots branching regulation, WOX11 ortholog was shown to be a part of WOX-CLAVATA regulatory loop with the *PgCLE45* gene. *PgCLE45* is induced directly by WOX11, and its product negatively impacts *WOX11* expression [6]. Thus, WOX11 is suggested to be a useful molecular tool for promoting the rooting of leaf or stem cuttings and enlargement of the root system in response to stressful environmental conditions [10]. However, it is worth noting that WOX11 orthologs in different species have quite specified functions and features. For example, in an epiphytic orchid species *Dendrobium catenatum*, aerial roots—a specialized type of adventitious root—are formed. At the same time, overexpression of *D. catenatum WOX12* in *A. thaliana* leads to the inhibition of root and shoot growth; leaf chlorosis; and, in general, promotes cell dedifferentiation and callus formation [11]. Such effects are distinct from the *A. thaliana WOX11* overexpression effect and suggest diversification of members of the WOX11/12 branch in different species.

The role of the WOX11 transcription factor is not limited to participation in rooting processes. In Hybrid Sweetgum (*Liquidambar styraciflua* × *L. formosana*) tissue cultures, *WOX11* ortholog is highly expressed during somatic embryogenesis (SE) and is almost not expressed in non-embryogenic calli or vegetative organs [12]. *MtWOX11-1* was also shown to be expressed specifically during somatic embryogenesis (SE) in *Medicago truncatula* [13]. Transcriptomic analysis of *A. thaliana* seeds revealed the participation of WOX11 in the regulation of seed dormancy and germination [14]. In rice, WOX11 interacts with SLENDER GUY2 and regulates grain width [15].

Proteins from the WOX8/9 branch are considered as regulators of the early stages of zygotic embryogenesis. *WOX8* is coexpressed with *WOX2* in the early zygote during zygotic embryogenesis in *A. thaliana* [16]. The activation of *WOX9* expression takes place during the two-cell stage. Later, in the octant embryo, all three genes are involved in maintaining of the patterning of the growing plant [16,17]. The loss of *WOX8* and *WOX9* function leads to patterning defects in the suspensor, resulting in death [18].

The role of *WOX8/9* genes in the SE is also known. High expression levels of *WOX9* homologs were observed in embryogenic or pro-embryogenic calli in *Vitis vinifera* [19], Hybrid Sweetgum (*Liquidambar styraciflua* × *Liquidambar formosana*) [12], and in *Dimocarpus longan* [20]. In *Medicago truncatula*, *MtWOX9-1* overexpression can stimulate SE [21]. However, in *Nicotiana tabacum*, only coexpression of *WOX2* and *WOX8* or *WOX9* leads to regeneration improvement, while increased expression of any of these three genes alone does not affect the explant phenotype [22].

Besides, WOX9 homologs also regulate development of diverse organs and tissues in different species. In *M. truncatula* and *N. sylvestris*, MtWOX9 and NsWOX9, respectively, act as abaxial factors, functioning antagonistically to WOX1 orthologs in the regulation of leaf blade development [23]. For the *Triticum aestivum WOX9* ortholog, specific expression in roots was shown [24,25], whereas its overexpression stimulated root system development in *A. thaliana* [24]. In *Solanaceae*, members of the WOX8/9 branch have a specific function related to the inflorescence development. For example, in *Solanum lycopersicum*, the *slwox9* mutant shows excessive branching of inflorescence meristems [26]. Loss of function of the *EVERGREEN* gene (*EVG*, *WOX9* homolog) in the *bonsai* mutant of *Petunia hybrida* leads to abnormalities in flower formation, inflorescence branching, and control of meristem size [27]. *P. hybrida* also has another *WOX9* homolog: *SISTER OF EVG* (*SOE*) [28]. Unlike *EVG*, *SOE* is expressed mainly during embryogenesis, in vegetative meristems, and in ovaries, demonstrating an expression pattern similar to *WOX9* [28].

Thus, roles of the WOX transcription factors from the intermediate clade can vary significantly depending on plant species and expression pattern. Nevertheless, they usually act as expression activators, and mechanisms of their functioning can be basically similar even for members of different subclades. For example, it was shown in *N. sylvestris* that NsWOX9 binds to the *NsCYTOKININ OXIDASE 3* promoter, acting as a transcriptional activator [29]. Thus, NsWOX9 can promote cytokinin degradation. Interestingly, WOX11 ortholog in rice was also recently shown to directly activate expression of *CKX4*, thereby stimulating crown root development [9]. In *A. thaliana*, WOX9 interacting with ARF5 (also known as MONOPTEROS, MP) in hypophysis takes part in primary root initiation, while the WOX11-ARF6/8 complex regulates the fate of an adventitious root founder cell [5].

A lot of functions of intermediate *WOX* genes are related with regeneration and embryogenesis. For some of them, the ability to stimulate regeneration was shown [21,22]. Therefore, we decided to check if other members of this clade can stimulate SE and be used to alleviate transformation. Since leguminous plants have low transformation effectiveness, which is probably related with poor regeneration capacity, we used a model leguminous plant *M. truncatula* and its genetically close lines differing in their capacity for SE.

## 2. Results

### 2.1. Analysis of the Intermediate Clade WOX Genes in the M. truncatula Genome

**Figure 1:**
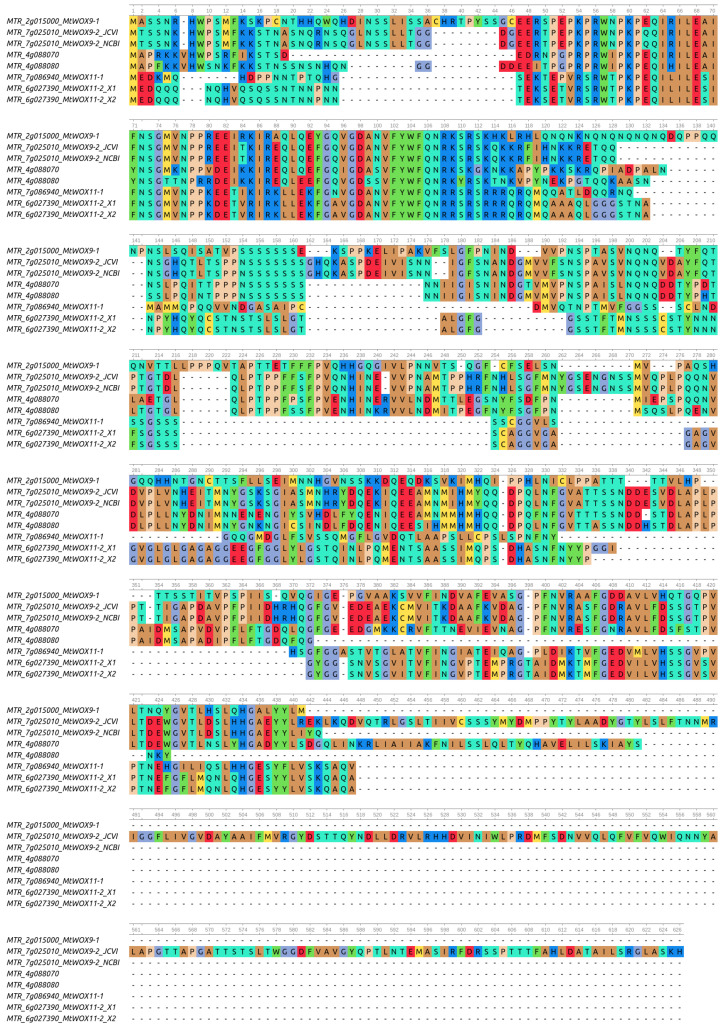
Alignment of *Medicago truncatula* WOX proteins from intermediate clade. Blue color marks basic amino acid residues, red color—acidic, brown color—hydrophobic, cyan—hydrophilic, yellow—sulfur-containing amino acid residues, violet marks glycine, and pink marks proline residues. MTR_7g025010_JCVI_annot—MtWOX9-2 protein sequence according to JCVI annotation Mt4.0v2 [32]. MTR_7g025010_MtWOX9-2_NCBI_annot—MtWOX9-2 protein sequence according to the NCBI *Medicago truncatula* Annotation Releases 101 and 102. MTR_6g027390_MtWOX11-2 X1 and 2—two isoforms of MtWOX11-2 protein obtained due to alternative splicing, according to the NCBI annotations 101 and 102.

**Figure 2:**
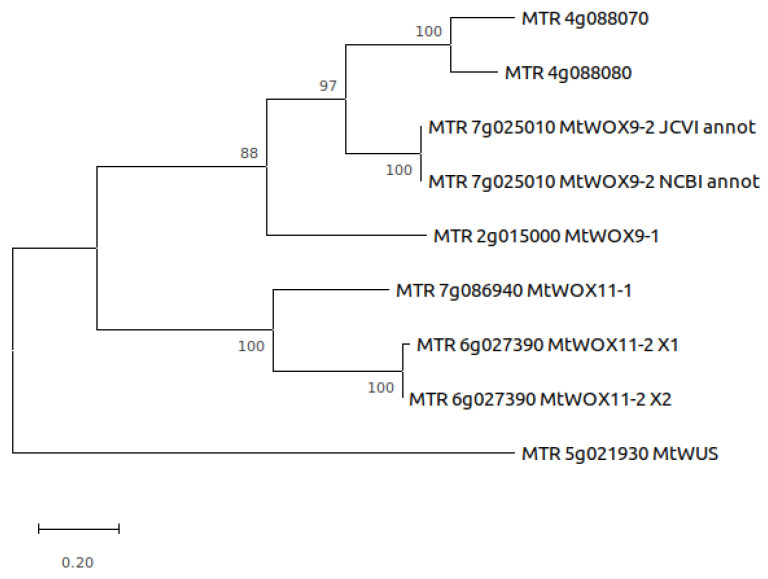
Phylogenetic tree of *M. truncatula* WOX proteins from intermediate clade, based on their full sequences. MTR_7g025010_JCVI_annot—MtWOX9-2 protein sequence according to JCVI annotation Mt4.0v2 [32]. MTR_7g025010_MtWOX9-2_NCBI_annot—MtWOX9-2 protein sequence according to the NCBI *Medicago truncatula* Annotation Releases 101 and 102. MTR_6g027390_MtWOX11-2 X1 and 2—two isoforms of MtWOX11-2 protein obtained due to alternative splicing, according to the NCBI annotations 101 and 102. The tree was inferred using the Maximum Likelihood method and JTT matrix-based model [33]. The percentage of trees in which the associated taxa clustered together is shown next to the branches. The tree is drawn to scale, with branch lengths measured in the number of substitutions per site. Evolutionary analyses were conducted in MEGA X [34].

The last genome version of *M. truncatula* [30] contains eighteen *WOX* genes, including six genes from the intermediate clade [31]. We aligned proteins encoded by these six genes with MUSCLE (Figure 1) and built a phylogeny tree using their full sequences (Figure 2). According to this tree, MtWOX9-1, which was previously shown to stimulate SE [21], has three closest paralogs. Those include MtWOX9-2 and two proteins, probably encoded by recently duplicated genes, *MTR 4g088070* and *MTR 4g088080*. Another two proteins, MtWOX11-1 and MtWOX11-2, are more distantly related to MtWOX9-1.

Among all these genes, specific expression during SE was shown for *MtWOX11-1* and *MtWOX9-1* [13]. *STENOFOLIA* and *MtWOX9-1*, two *WOX* genes with specific expression in the embryogenic calli, were shown to stimulate SE when overexpressed [21,35]. We supposed that *MtWOX11-1* overexpression could also stimulate SE in calli. *MtWOX9-2* and *MtWOX11-2* are closely related to *MtWOX9-1* and *MtWOX11-1*, respectively. We evaluated their expression during SE using the same system that was used for expression analysis of *MtWOX11-1* and *MtWOX9-1*, including calli of embryogenic line 2HA and non-embryogenic line A17 [13]. *MtWOX9-2* mRNA was detected at different cultivation stages in different lines, but only in single technical and biological replicates. Thus, we did not obtain stable reproducible results on the *MtWOX9-2* mRNA presence. These results suggest that *MtWOX9-2* has very low and/or unstable expression, which cannot be detected with usual qPCR.

qPCR analysis of *MtWOX11-2* expression produced some unexpected results. At first, we used qPCR primers annealing on the 3′ part of *MtWOX11-2* cds (Figure S1A). According to the qPCR, *MtWOX11-2* mRNA was not detected in any of the samples. The primers gave PCR product of corresponding size when used with genomic DNA of *M. truncatula;* therefore, they should be functional in qPCR with cDNA. However, according to the transcriptomic analysis, *MtWOX11-2* had a rather high expression level in the embryogenic calli [36]. At the same time, *MtWOX11-2* mRNA is known to have at least two different isoforms that differ in their 3′ parts. Given this information, we used another primers pair that annealed on the 5′ part of *MtWOX11-2* cds (Figure S1A). According to the qPCR with these primers, *MtWOX11-2* did not have any specific expression pattern in embryogenic or non-embryogenic calli, but demonstrated a stable expression level during in vitro cultivation (Figure S1B). Therefore, there is a possibility of presence of some specific *MtWOX11-2* isoform in the calli of A17 and 2HA lines.

### 2.2. MtWOX9-1 and 2 Differ in Their Ability to Stimulate SE in the Embryogenic Line

We compared the effect of MtWOX9-1 and MtWOX9-2 on SE capacity of the embryogenic line (R108). For *MtWOX9-1* overexpression, we used a vector construction obtained previously [21,23]. To obtain a vector for *MtWOX9-2* overexpression, we tried to amplify *MtWOX9-2* cds from cDNA, obtained from seeds, flowers, shoot apices, or embryogenic calli. For amplification, we used primer sequences designed according to the JCVI annotation Mt4.0v2 and NCBI *Medicago truncatula* Annotation Releases 101 or 102; however, none of these primers gave a product, probably due to the low expression level of *MtWOX9-2*. Since MtWOX9-2 still could be a potential SE stimulator, we used the chemically synthesized cds of this gene. It should be noted that we chose the MtWOX9-2 protein sequence derived from JCVI annotation Mt4.0v2 [32] because it is more distant from the MtWOX9-1 protein than MtWOX9-2 sequences derived from other annotations. We supposed that such a protein variant could exhibit some new effects on transgenic callus development.

**Figure 3:**
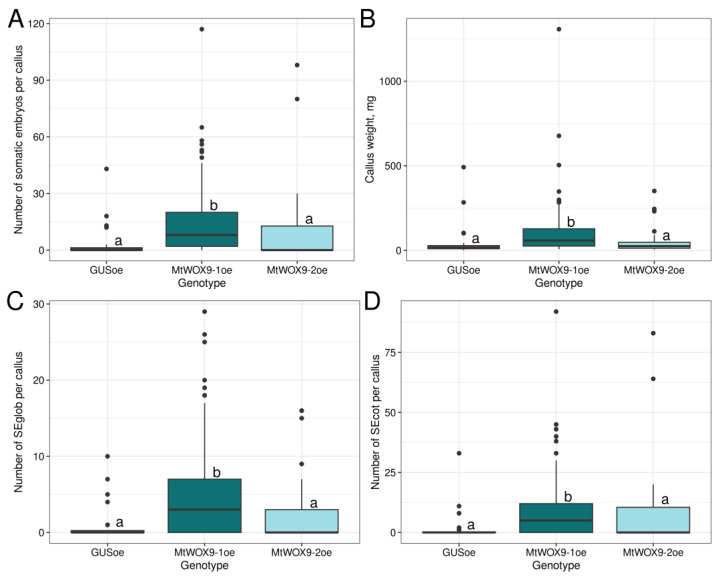
Boxplots representing number of somatic embryos per callus (**A**), weight (**B**), number of globular somatic embryos (SEglob) per callus (**C**), and number of somatic embryos with cotyledon primordia (SEcot) per callus (**D**) for calli, obtained after explant transformation with constructions for *GUS*, *MtWOX9-1*, or *MtWOX9-2* overexpression. Data are obtained from 28–113 calli for different samples. To assess the statistical significance of the observed differences, the Dunn test with Holm *p*-value adjustment was used. Different lowercase letters represent values with statistically significant differences (*p*-value < 0.05). Analysis of *M. truncatula* SE capacity and weight measurement of T0 calli were performed on the 79th day after transformation, after 49 days of cultivation on the callus induction medium and 30 days of cultivation on the hormone-free medium.

We transformed leaf explants with constructions for overexpression of *MtWOX9-1*, *MtWOX9-2*, or with control construction for *GUS* overexpression. After about 60 days of cultivation, transgenic calli were developed, and we evaluated their weight and number of somatic embryos per callus. As in our previous research [21], calli with *MtWOX9-1* overexpression formed more somatic embryos than control calli (Figure 3A). The same increase in somatic embryo number persisted when we evaluated only the number of globular somatic embryos (SEglob) or somatic embryos with cotyledon primordia (SEcot) (Figure 3C,D). *MtWOX9-1* overexpressing calli also had increased weight (Figure 3B). At the same time, calli with *MtWOX9-2* overexpression did not demonstrate significant differences in SE capacity or weight in comparison with control (Figure 3). Together, these data allowed us to suppose that effects of overexpression of these two closely related genes differ between each other.

### 2.3. MtWOX9-1 Can Induce SE in the Non-Embryogenic Line, Unlike MtWOX9-2

**Figure 4:**
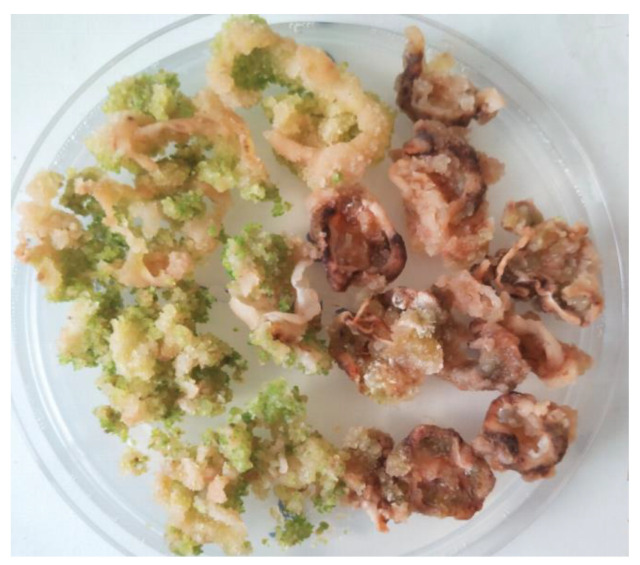
Non-transgenic calli of R108 (**left**) and 108-1 lines (**right**) after 30 days of cultivation on the callus induction medium and 30 days of cultivation on hormone-free medium.

We also had a non-embryogenic progenitor of R108, the 108-1 line [37]. In our cultivation system, which is used to induce SE in the R108 line, 108-1 explants also form calli, although they are smaller. We did not detect somatic embryos on such calli (Figure 4).

We supposed that MtWOX9-1 may not only increase SE capacity, but also induce SE in this non-embryogenic line. Assuming that processes that increase SE capacity and processes that induce SE may have different mechanisms, we also decided to check if *MtWOX9-2* overexpression is capable of inducing SE in the non-embryogenic line.

**Figure 5:**
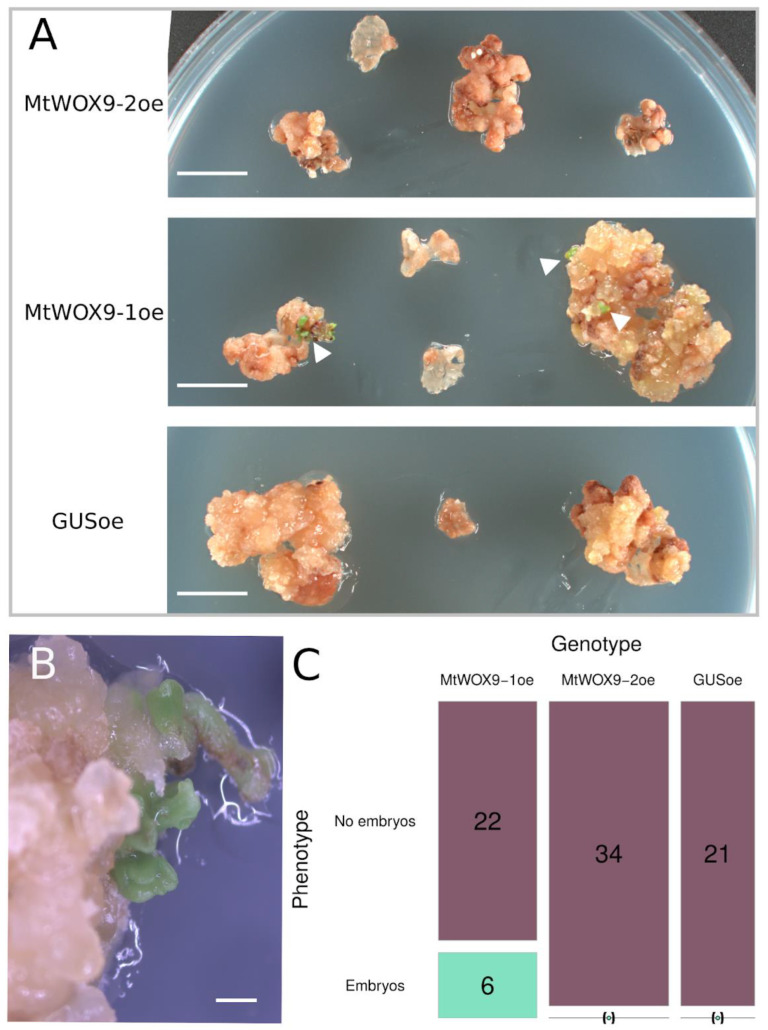
(**A**) Calli obtained as a result of 108-1 line leaf explants transformation with constructions for overexpression of *MtWOX9-2* (**upper part**), *MtWOX9-1* (**middle part**), and *MtWOX9-2* (**bottom part**). Somatic embryos are indicated with white arrowheads. Scale bars are 1 cm. (**B**) Somatic embryo development in 108-1 calli with *MtWOX9-1* overexpression. Scale bar is 1 mm. (**C**) Mosaic plot representing number of explants that formed somatic embryos after transformation with constructions for *MtWOX9-1*, *MtWOX9-2*, and *GUS* overexpression. Explants count and photo taking were performed on the 77th day after transformation, after 42 days of cultivation on the callus induction medium and 35 days of cultivation on the hormone-free medium.

According to our results, 108-1 explants transformed with the construction for *MtWOX9-1* overexpression could indeed form somatic embryos. In two experiments performed, six out of twenty-eight explants (21%) (Figure 5) and sixteen out of forty explants (40%) (Figure S2) formed somatic embryos. At the same time, among control explants transformed with the construct for *GUS* overexpression (58 explants in total for two experiments), only a single explant formed somatic embryos. Transformation of 108-1 explants with the construct for *MtWOX9-2* overexpression did not have any noticeable effect on these explants (Figure 5). Thus, MtWOX9-1 has the ability to induce SE in the non-embryogenic line, whereas for MtWOX9-2, no significant impact on calli development was detected in the embryogenic or in the non-embryogenic line.

### 2.4. MtWOX11-1 and 2 Do Not Stimulate SE in the Embryogenic Line

**Figure 6:**
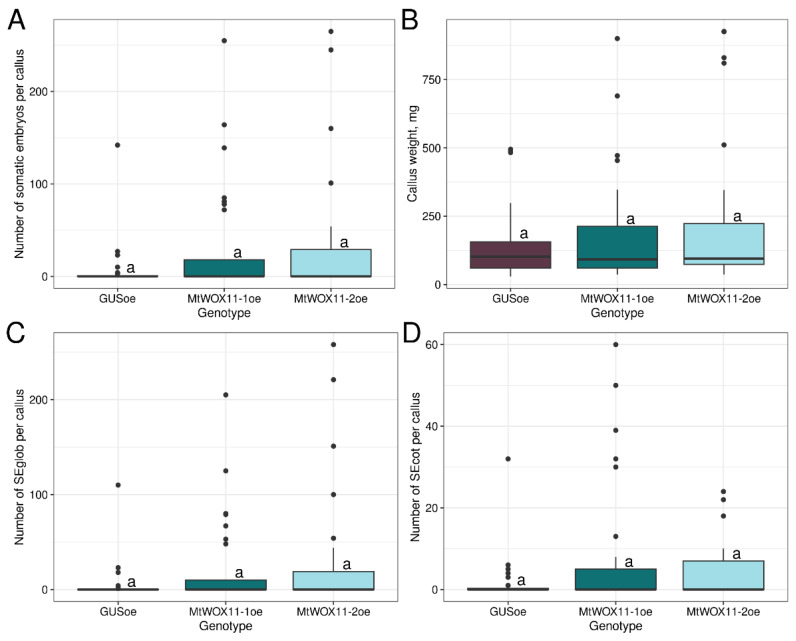
Boxplots representing number of somatic embryos per callus (**A**), weight (**B**), number of globular somatic embryos (SEglob) per callus (**C**), and number of somatic embryos with cotyledon primordia (SEcot) per callus (**D**) for calli, obtained after explant transformation with constructions for *GUS*, *MtWOX11-1*, or *MtWOX11-2* overexpression. Data are obtained from 24–29 explants for different samples. To assess the statistical significance of the observed differences, the Dunn test with Holm *p*-value adjustment was used. Different lowercase letters represent values with statistically significant differences (*p*-value < 0.05). Analysis of *M. truncatula* SE capacity and weight measurement of T0 calli were performed on the 77th day after transformation, after 42 days of cultivation on the callus induction medium and 35 days of cultivation on the hormone-free medium.

As our next step, we performed a similar analysis with both *M. truncatula* genes from the WOX11/12 branch—*MtWOX11-1* and *2*. These genes were cloned into pMDC32 vectors for overexpression. Plasmid sequencing showed that the *MtWOX11-2* cds corresponded to the X1 isoform. The effect of transformation with vectors for *MtWOX11-1* or *2* overexpression was evaluated in the obtained T0 calli. As a result, we did not find significant differences between calli with constructions for *MtWOX11-1* or *2* genes overexpression and control calli, neither in SE capacity nor in weight parameter (Figure 6). Thus, these two genes do not have the same SE stimulating activity as *MtWOX9-1*.

## 3. Discussion

Stimulation of regeneration in transformed explants is an important challenge for plant genetic engineering and often for plant genome editing. One of possible pathways to achieve regeneration is the usage of morphogenic regulators. These genes are sufficient to induce SE or shoot development when ectopically expressed in transformed cells. WUSCHEL is probably the most well-known morphogenic regulator, and it can be used to alleviate transformation of many species including grasses and some dicots [38,39]. Members of the WOX family are very divergent in their functions; at the same time, most of them regulate similar processes, such as cell division and differentiation. Thus, besides WUS, other WOX proteins may also be revealed as potent morphogenic regulators that can be used for plant transformation. For example, the *WOX5* gene is known to be expressed in the quiescent center and maintain the root apical meristem, activating local auxin synthesis [40]. At the same time, it is a very important participant of callus formation, shoot regeneration, and SE [41,42]. Similarly to its functioning in root, it activates auxin synthesis in a specific cell layer in the callus and also increases cytokinin sensitivity. This is supposed to induce acquisition of cell pluripotency, which is necessary for further regeneration [42]. Indeed, the WOX5 ortholog in *Triticum aestivum* was shown to induce shoot regeneration, alleviating transformation of this species [43]. In *M. truncatula*, not only WUS [44], but also STENOFOLIA (MtWOX1) and MtWOX9-1 stimulate SE [21,35]. To find new SE stimulators among *WOX* genes in *M. truncatula*, we decided to analyze different members of the intermediate clade, because they are usually involved in regeneration and embryogenesis. As in our previous research, *MtWOX9-1* overexpression stimulated SE in the embryogenic R108 line. More importantly, *MtWOX9-1* overexpression was able to induce SE in non-embryogenic 108-1 line. Thus, MtWOX9-1 probably is one of key SE regulators, whose ectopic expression is sufficient to start SE.

In *N. tabacum* leaf explants, coexpression of *A. thaliana WOX2* and *WOX8* or *9* could induce regeneration, whereas overexpression of either of these genes alone could not [22]. These data suggest that *A. thaliana* WOX9 does not have a regeneration inducing ability like MtWOX9-1. It would be intriguing to compare the effects of *A. thaliana WOX9* and *MtWOX9-1* in *Medicago truncatula* explants.

At the same time, calli with *MtWOX9-2* overexpression in the embryogenic R108 line had some tendency to form more somatic embryos, which probably could be demonstrated clearly if more explants were analyzed. But such an impact, if it exists, is undoubtedly weaker than the overexpression effect of *MtWOX9-1*. Therefore, MtWOX9-2 is less likely to be used as a morphogenic regulator. Previously, *MtWOX9-2* overexpression was shown to cause changes in leaf morphology in *N. tabacum*. In that study, the cds sequence according to the NCBI annotation was used [23], whereas we used the cds sequence designed according to the JCVI annotation. It would be interesting to check if the cds sequence used in our research is able to change leaf morphology being overexpressed in tobacco, and, vice versa, if the cds designed according to NCBI annotation can induce SE when overexpressed in *M. truncatula*.

The *MtWOX11-1* or *2* overexpression did not have any significant impact on embryogenic calli development in our cultivation system. So far, no data on WOX11 orthologs inducing SE or shoot regeneration have been published; however, they were shown to induce rooting and callus development [10]. On the other hand, *MdWOX11* overexpression was shown to repress shoot regeneration in *Malus domestica* [7]. Since the expression of *MtWOX11-1* increases during SE [13], it prompts finding the functions of this gene in this process, probably using mutant analysis.

Other two closely related genes from the intermediate WOX branch, *MTR_4g088070* and *MTR_4g088080*, have not been analyzed yet. According to the phylogenetic tree, they are more closely related with *MtWOX9-2* than *MtWOX9-1*; however, based on the alignment, they have several significant differences from both *MtWOX9-1* and *2*. Thus, the effect of these two genes on SE should also be checked.

Together, our data confirm the possibility of MtWOX9-1 use as a regeneration inductor in *M. truncatula*. That can be helpful for designing the optimal variant of morphogenic regulator for other legumes, most of which are recalcitrant to transformation. Our data also demonstrate the specialized functions of *WOX* genes from the intermediate clade: out of 4 checked genes, only *MtWOX9-1* overexpression stimulated SE. Besides, even genes within the same subclade, like *MtWOX9-1* and *MtWOX9-2*, differed in their effects. It is also worth noting that these differences cannot be explained by different expression patterns, because the effects were evaluated for ubiquitous ectopic expression of genes due to the 35S promoter. Thus, we can suppose that combinations of different WOX family members with different in vitro conditions (for example, with various exogenous hormone concentrations) can be used to override various limiting steps for regeneration and transformation in diverse recalcitrant plant species.

## 4. Materials and Methods

### 4.1. Plant Material

For evaluation of the effect of different *WOX* genes overexpression, *M. truncatula* non-embryogenic 108-1 line and embryogenic R108 line were used [37]. Seeds of the R108 line were provided by the Samuel Roberts Institute (Ardmore, OK, USA). Seeds of the 108-1 line were provided by Dr Pascal Ratet (Paris-Saclay University, Gif-sur-Yvette, France). The 108-1 line calli are developed from leaf explants mostly during cultivation on the hormone-containing medium for callus induction. When they are transferred onto the SE-inducing hormone-free medium, they apparently stop their development. We could not isolate RNA from 108-1 calli cultivated more than 10 days on the hormone-free medium, which allows to suppose that they are dead at this stage. Therefore, the use of R108 and 108-1 lines for expression analysis dynamics is inconvenient. For the evaluation of the dynamics of gene expression, *M. truncatula* A17 and 2HA lines were used. Seeds of the 2HA line were provided by Dr. Mireille Chabaud (French National Institute for Agriculture, Food, and Environment, Paris, France). Seeds of the A17 line were provided by Wageningen University (Wageningen, The Netherlands). Similarly to R108 and 108-1 lines, the 2HA line is a descendant of the A17 line obtained after multiple rounds of in vitro cultivation and selection of regenerants with high SE capacity. The 2HA and A17 lines are supposed to be nearly isogenic, and they differ from each other mainly in their SE capacity [45]. In our cultivation system, during 6 weeks of cultivation, leaf explants from both of them develop viable calli, but 2HA calli form somatic embryos, whereas A17 calli do not. Therefore, these two lines allow us to evaluate gene expression dynamics during both embryogenic and non-embryogenic callus development.

### 4.2. Strains of Microorganisms

To obtain genetic constructs, we used the *Escherichia coli* strain DH10B [46]. *E. coli* bacteria were grown in standard cultivation conditions [47]. Transformation of *E. coli* was performed using the standard protocol with calcium chloride [48]. *R. radiobacter* bacteria were grown in solid or liquid YEP medium (for 1 l: 5 g NaCl, 10 g tryptone, 10 g yeast extract, and 15 g agar (in case of solid medium)). Transformation of *R. radiobacter* was performed by freeze–thaw method [49].

### 4.3. Phylogenetic Analysis

For protein alignment, the MUSCLE algorithm [50] and the MEGA X software (version 10.0.5) [34] were used. Alignment visualization was obtained with Ugene [51]. The phylogenetic tree of *M. truncatula* WOX proteins was built in the MEGA X software [34] using the Maximum Likelihood method and JTT matrix-based model [33].

### 4.4. Expression Analysis

For sequence analysis and primer design, Primer 3 (accessed on October 2022) [52], Ugene version 48.1 [51], SnapGene (from GSL Biotech; available at snapgene.com, accessed on 8 November 2023), and ApE (M. Wayne Davis) were used. RNA isolation and qPCR analysis were performed as described in [53]. Primers for qPCR for *MtH3L* and *MtWOX9-2* genes were taken from [23,54], respectively. The *MtH3L* gene (*Medtr4g097170*) was used as a reference because it demonstrated stable expression in calli in our previous studies [53].

### 4.5. Vectors Construction

The vector for *MtWOX9-1* overexpression was obtained previously [23]. *MtWOX11-1* and *2* cds were amplified by PCR on a mixture of cDNAs obtained from *M. truncatula* pods (11 days after anthesis) and seeds (11 days after anthesis). *MtWOX11-2* cds was amplified by PCR on a mixture of cDNAs obtained from *M. truncatula* pods, seeds, and calli with somatic embryos. Matrix for amplification of *MtWOX9-2* cds was synthesized by the Evrogen company (Moscow, Russian Federation). The cds fragments were isolated from the agarose gel using the Cleanup Mini kit (Evrogen (Moscow, Russian Federation)). The Gateway method [55] was used to obtain vectors. The pDONR207 vector was used as a donor plasmid. For overexpression, pMDC32 destination plasmid was used, in which cds of the gene of interest was put under the control of double 35S promoter [56]. Primers used in the study are presented in Table S1.

### 4.6. Plant Growing Conditions

Plants were grown in a room with artificial light at a temperature of 21–23 °C with 16/8 light/dark regime. The following germination protocol for *M. truncatula* was used: seeds were treated with concentrated sulfuric acid (95%) for 10 min, then washed 10 times with sterile distilled water. The seeds were then placed on agar plates (1%) and incubated at 4–8 °C for 7–14 days until germination started. According to our observations, such prolonged incubation in low temperatures slowed down the root growth in germinated seedlings and simultaneously stimulated germination of dormant seeds, allowing us to synchronize seedlings growth. Then, agar plates with seedlings were placed in RT in darkness for one night; after that, seedlings were transplanted into soil (approximately 50% Terra Vita universal soil and 50% vermiculite) or on the modified Fahraeus medium [21,57].

### 4.7. Obtaining Transgenic Calli and Evaluation of SE Capacity

**Agrobacterium preparation.** Before the start of transformation, agrobacterium strain AGL1 with the required plasmid was sown on YEP solid selective medium with antibiotics (rifampicin, kanamycin) and incubated at 30 °C for approximately 24 h. A small number of bacteria from this solid medium were then inoculated into liquid YEP (5–10 mL) with the required antibiotics and allowed to grow overnight at 30 °C on a shaker (200 rpm). The next day, 2 mL of the overnight culture were diluted in 30 mL of liquid YEP medium or in the AB-MES medium [58] with 200 mkM acetosyringone. Both variants of media also included 40 mg/L rifampicin and 50 mg/L kanamycin. Bacteria were grown for several hours until the optical density OD600 = 0.5–1.0. The culture was then pelleted, the liquid was decanted, and the sediment was resuspended in the infiltration medium so that the final optical density of agrobacteria was about 0.4–0.6 optical units and was equal for all constructs analyzed in given experiment. Depending on the experiment, different variants of infiltration medium were used: standard variant (0.5X modified liquid PCI-4 medium [37] with 18 µM 2,4-D; 2.22 µM BAP) or modified variant (0.5X AB-MES components and 0.5X modified liquid PCI-4 medium [37] components with 18 µM 2,4-D; 2.22 µM BAP; and 200 mkM acetosyringone).

**Explants preparation.** As a source of leaf explants, 30–45 day old soil-grown *M. truncatula* plants were used. For sterilization, *M. truncatula* leaves were placed in falcon tubes with distilled water and then incubated in 70% ethanol for 1 min. After ethanol removal, leaves were submerged in 1–3% NaOCl solution (10 times diluted Belizna bleach) with 1–2 drops of Tween20 and incubated with nutation for 10 min. Then, the leaves were rinsed with sterile distilled water 5–7 times. In some experiments, 25–30 day old sterile plants grown on the modified Fahraeus medium were used as the leaf explants source. In that case, sterilization and rinsing steps were skipped. For explant preparation, 3 simple leaflets were cut from each sterile leaf with a scalpel. Small cuts perpendicular to the leaflet main vein were also made on the leaflet abaxial side with a scalpel.

**Infiltration and cocultivation.** In the next stage, the leaflets were placed in falcon-type tubes with an agrobacterium culture in the infiltration medium and incubated for 15 min with nutation. The infiltration medium was then discarded and the leaves were placed on the standard solid cocultivation medium (modified PCI-4 medium [37] with 18 µM 2,4-D and 2.22 µM BAP), or on the modified solid cocultivation medium, which was identical to the modified variant of infiltration medium, except that phytagel (6 g/L) was added for solidification. Explants were incubated for approximately 40 h in the dark.

**In vitro cultivation.** After cocultivation, explants were transferred onto the solid SH medium for callus formation (modified PCI-4 medium [37] with 18 µM 2,4-D; 2.22 µM BAP; and with the addition of two selective agents: cefotaxime (250 mg/L), to kill agrobacteria, and hygromycin (25 mg/L), necessary for the selection of transgenic plant cells). The explants were cultured on this medium in the darkness for 30–40 days. Every 10–14 days, they were put on a fresh medium. Then, the resulting calli were transplanted onto the SE-inducing medium—a modified PCI-4 medium [37], which did not contain hormones, with the addition of cefotaxime (250 mg/L) and hygromycin (12.5 mg/L). They were cultivated on such medium for a month, with a transfer to a fresh medium every 10–14 days.

**SE capacity evaluation.** Embryos were counted using stereoscope after cultivation on a hormone-free medium at the 77–79th day after explants infiltration. The number of globular somatic embryos (SEglob) and somatic embryos with visible cotyledon primordia in the heart, torpedo, and cotyledonary stage (SEcot) was counted separately. The SEcot often formed fused inseparable structures [21]. Every inseparable structure containing at least one somatic embryo with cotyledon primordia was counted as one SEcot. Counting of somatic embryos was performed using a blind procedure, i.e., the researcher did not know the genotype of callus analyzed.

### 4.8. Data Analysis

Statistical analysis and diagram drawing were performed in the R environment [59] using Rstudio [60]. R packages rstatix [61], ggpubr [62], dplyr [63], rtables [64], formatters [65], ggplot2 [66], patchwork [67], rcompanion [68], fastDummies [69], multcompView [70], purrr [71], and stringr [72] were used.

## Data Availability

Data is contained within the article and Supplementary Materials.

## Supplementary Materials

The following supporting information can be downloaded at: https://www.mdpi.com/article/10.3390/plants13020223/s1, Table S1. Primers used in the study. Gene IDs in tables correspond to the versions MedtrA17_4.0 [32] and MtrunA17r5.0-ANR [30] of *M. truncatula* genome; Figure S1. (A) The map of *MtWOX11-2* X1 cds. First variant of primers (MtWOX11-2_rt1 for and rev) and second variant of primers (MtWOX11-2 rt2 for and rev) positions are shown on the cds. (B) *MtWOX11-2* gene expression dynamics during in vitro cultivation of explants of embryogenic 2HA (light blue) and non-embryogenic A17 (brown) lines. Expression was evaluated with qPCR with MtWOX11-2 rt2 primers. Error bars represent the standard error. Data are obtained from three biological replicates. To assess the statistical significance of the observed differences, one-way analysis of variance (one-way ANOVA) with Tukey’s post hoc test was used, with confidence level 0.95. Different lowercase letters represent expression levels with statistically significant differences (*p*-value < 0.05); Figure S2. Mosaic plot representing number of explants that formed somatic embryos after transformation with constructions for *MtWOX9-1* and *GUS* overexpression. Explants count was performed on the 82nd day after transformation, after 41 days of cultivation on the callus induction medium and 41 days of cultivation on the hormone-free medium.
