# Supplementary figures and images for: The WOX Genes from the Intermediate Clade: Influence on the Somatic Embryogenesis in Medicago truncatula
Source: Plants (Basel). 2024 Jan 13;13(2):223. doi: 10.3390/plants13020223 (PMC10819790; doi:10.3390/plants13020223)

A

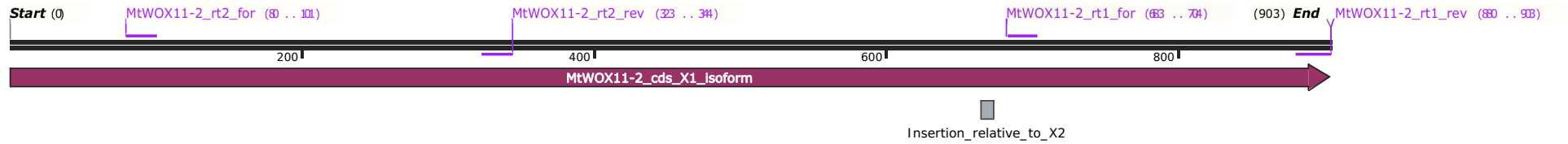

B

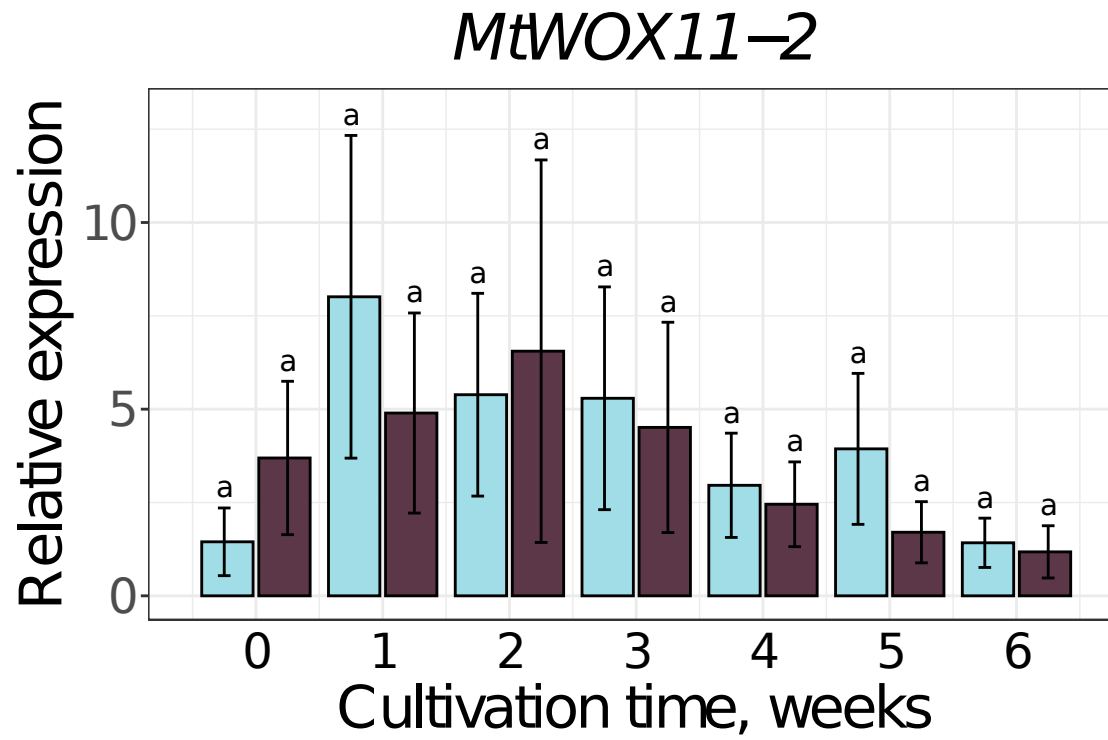

Supplement: Supplementary file 1 [file plants-13-00223-s001.zip › Fig_S1.pdf]

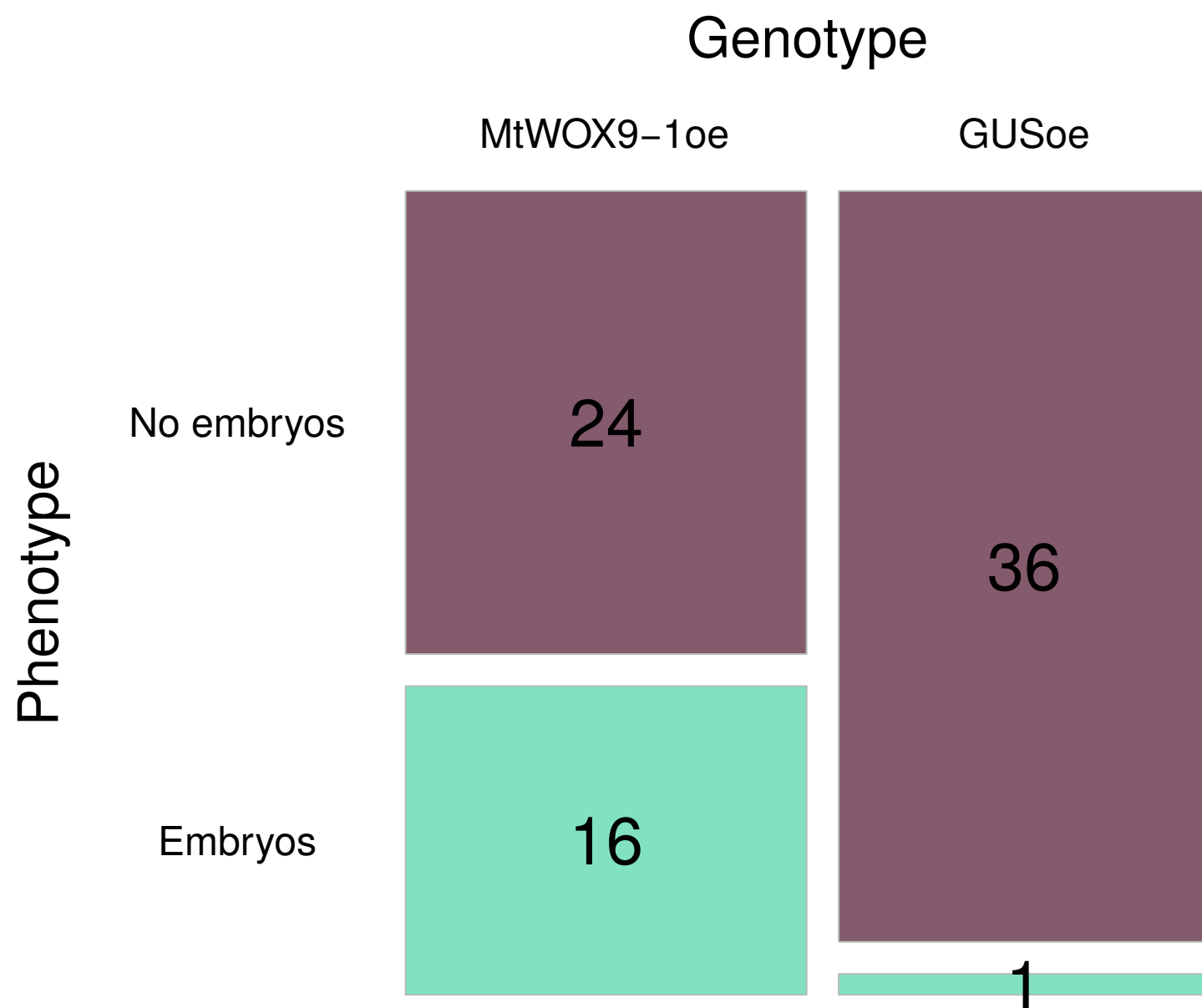

Supplement: Supplementary file 1 [file plants-13-00223-s001.zip › Fig_S2.pdf]
